# The Challenges and Effects of Ascorbic Acid Treatment of Acute Pancreatitis: A Systematic Review and Meta-Analysis of Preclinical and Clinical Studies

**DOI:** 10.3389/fnut.2021.734558

**Published:** 2021-10-26

**Authors:** Lin Gao, Eric Chong, Sayali Pendharkar, Anthony Phillips, Lu Ke, Weiqin Li, John Albert Windsor

**Affiliations:** ^1^Department of Critical Care Medicine, Center of Severe Acute Pancreatitis (CSAP), Jinling Hospital, Medical School of Nanjing University, Nanjing, China; ^2^Applied Surgery and Metabolism Laboratory, School of Biological Sciences, University of Auckland, Auckland, New Zealand; ^3^Faculty of Medical and Health Sciences, Surgical and Translational Research Centre, School of Medicine, University of Auckland, Auckland, New Zealand

**Keywords:** acute pancreatitis, ascorbic acid, clinical outcomes, oxidative stress, pancreatic injury

## Abstract

**Background:** Oxidative stress has been implicated in the pathogenesis of acute pancreatitis (AP), and ascorbic acid (AA), as an important endogenous antioxidant substance, has been shown to reduce AP severity in preclinical studies. However, the effects of AA supplementation in clinical settings remain controversial.

**Methods:** PubMed, EMBASE, MEDLINE, and SCOPUS databases were searched, and both preclinical and clinical studies were included. For clinical trials, the primary outcome was incidence of organ failure, and for preclinical studies, the primary outcome was histopathological scores of pancreatic injuries.

**Results:** Meta-analysis of clinical trials showed that compared with controls, AA administration did not reduce the incidence of organ failure or mortality during hospitalization but was associated with significantly reduced length of hospital stay. Meta-analysis of preclinical studies showed that AA supplementation reduced pancreatic injury, demonstrated as decreased histological scores and serum amylase, lipase levels.

**Conclusion:** AA administration has no effect on survival or organ failure in patients with AP but may reduce the length of hospital stay. However, the evidence to date remains sparse, scattered, and of suboptimal quality, making it difficult to draw any firm conclusion on the clinical benefits of AA in AP.

## Introduction

Acute pancreatitis (AP) is a common inflammatory disease of the pancreas that carries a significant morbidity and mortality risk (1). About 80% of patients have mild AP and recover within a week with conservative treatment. The remaining 20% develop severe AP (SAP), defined by persistent organ failure, and is often associated with pancreatic necrosis and other local and systemic complications (2). The failure of drug trials in AP is well-documented, and there remains no specific and effective drug treatment (3).

Ascorbic acid (AA, vitamin C) is not only an essential nutrient involved in many metabolic pathways but also an important component in endogenous antioxidant defense. It functions as scavengers of reactive oxygen species (ROS), which damage cellular proteins, lipids, and DNA. As a cofactor for many enzymatic reactions, AA is involved in catecholamine, vasopressin, and steroid synthesis, as well as for carnitine and collagen production. Under optimal physiological conditions, dietary intake is sufficient to maintain AA levels within normal range (4). However, AA insufficiency may present in patients with acute and critical illness, including sepsis, trauma, and AP, which is associated with increased systemic oxidative stress and inflammation. Reduced intake of AA, increased tissue consumption, and high leukocyte turnover all contribute to this AA deficiency (5). The latter is important since intracellular AA concentrations in leucocytes are almost 80 times higher than in plasma (6), high production and turnover of leukocytes during the inflammatory response causes depletion (7).

Research to date shows that AP is associated with a high level of oxidative stress and that the markers of oxidative stress are significantly correlated with disease severity and recovery (8). In patients with AP, it has been shown that endogenous plasma's AA levels continue to decrease over the first 5 days after admission, and the degree of decrease is associated with disease severity (9). In view of the multiple and beneficial effects of AA and its decrease with the disease, restoring circulating AA levels is a plausible treatment strategy that might improve clinical outcomes and reduce morbidity and mortality. There are preclinical and clinical studies that provide evidence to support the use of AA as a novel adjuvant therapy in SAP (10) to reduce the risk of organ failure and mortality.

The aim of this study was to systematically review the effect of AA treatment on organ failure and mortality in patients with AP and on the severity of AP in animal models.

## Materials and Methods

This systematic review was prepared using the Preferred Reporting Items for Systematic Reviews and Meta-Analyses (PRISMA) guidance for literature review, extraction of data, and reporting of results (11).

### Search Strategy

Two investigators (LG and EC) independently searched PubMed, EMBASE, MEDLINE, and SCOPUS databases from their inception to September 5, 2020, for relevant studies. Abstracts from main international conferences and the reference lists of included studies were also screened for additional relevant studies. The following keywords or MeSH headings were used: “vitamin C” OR “ascorbic acid” OR “ascorbate” AND “acute pancreatitis.” Preclinical and clinical studies were included.

### Study Selection Criteria

The inclusion criteria of clinical trials were as follows:

1. Study population: adult patients (aged ≥18 years) with AP.
2. Intervention: AA as a monotherapy or in combination with other antioxidants.
3. Comparison: a placebo or no intervention.
4. Outcomes: hospital mortality, organ failure during hospitalization, and length of hospital stay.
5. Study design: parallel group randomized controlled trials (RCTs).

The exclusions for the clinical studies were the absence of a control arm, and RCTs conducted in patients with chronic pancreatitis or post-ERCP pancreatitis were excluded.

The inclusion criteria of preclinical studies were as follows:

1. Study subjects: animal models with AP.
2. Intervention: AA as monotherapy or in combination with other antioxidants.
3. Comparison: no intervention.
4. Outcomes: pancreatic injury based on physiologic or histologic measures.
5. Study design: comparative studies.

The exclusion criteria for the preclinical studies were the absence of histological scores or data on physiologic parameters. studies involving experiments conducted in animal models with chronic pancreatitis models were excluded.

### Data Extraction

Data from included studies were extracted by two independent authors (LG and EC) and discrepancies were resolved through discussion until consensus was reached. For clinical trials, the following data were extracted: name of the first author, year of publication, journal of publication, number of patients, study population, AA dose, treatment initiation and duration, routes of administration, and clinical outcomes. The primary outcome of interest was the incidence of organ failure during hospitalization, and the secondary outcomes were hospital mortality and length of hospital stay.

For preclinical studies, the following data were extracted: name of the first author, year of publication, journal of publication, animal models used, AA dose, treatment initiation and duration, routes of administration, and study outcomes. The primary outcome of interest was the histopathological scores of pancreatic injury and secondary outcomes were serum amylase and lipase levels.

### Assessment of Risk of Bias

For clinical trials, the Cochrane Collaboration tool was used to assess the risk of bias (12). Quality items assessed consisted of random sequence generation (selection bias), allocation concealment (selection bias), blinding of participants and personnel (performance bias), blinding of the outcome assessment (detection bias), incomplete outcome data (attrition bias), selective reporting (reporting bias), and other bias. Each item was assigned a low, unclear, or high risk of bias.

For preclinical studies, study quality and risk of bias assessment were based on the Systematic Review Center for Laboratory animal Experimentation (SYRCLE) grading system, which is an adapted version of the Cochrane risk of bias tool (13).

### Statistical Analysis

Data were analyzed using the Review Manager 5.3 software (The Nordic Cochrane Center, Copenhagen, Denmark). The results were presented as forest plots and odds ratios (ORs) with 95% CI for dichotomous data and mean difference (MD) or standardized MD (SMD) with 95% CI for continuous data. The *I*^2^-statistic was used to assess statistical heterogeneity among the studies in the meta-analyses. Values of *I*^2^ > 50% indicated moderate heterogeneity, and over 75% indicated a high level of heterogeneity. The Inverse-variance weighting method was used to combine independent continuous outcomes and a Mantel–Haenszel (MH) method to provide a pooled OR for dichotomous outcomes. If heterogeneity was observed (*I*^2^ ≥ 50%), the random effects model was applied; otherwise, a fixed effects model was used. Tests for publications, such as the funnel plot, were not carried out in this study in view of the low number of included studies. A *p* < 0.05 was considered statistically significant.

## Results

### Study Selection

**Figure 1:**
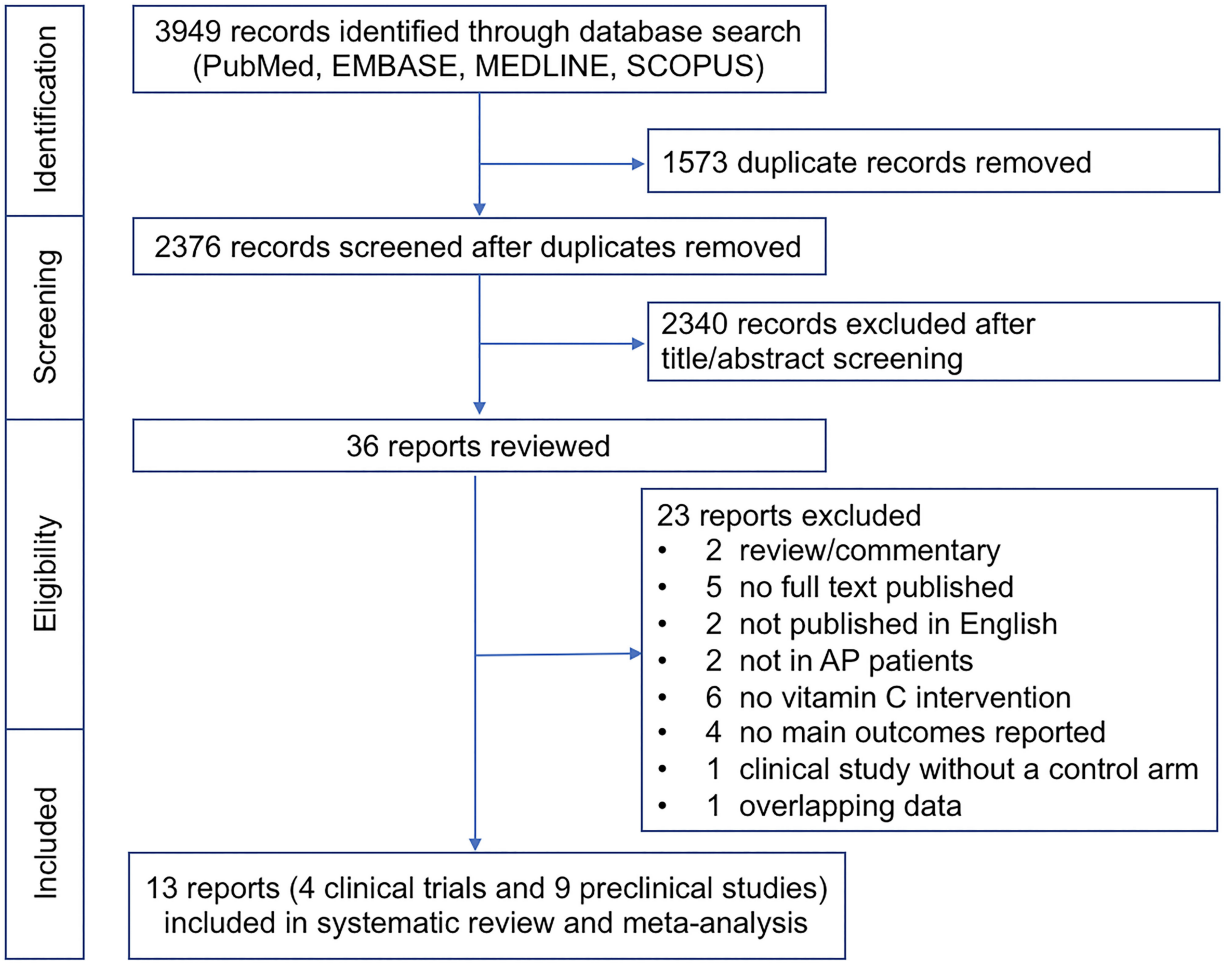
Flow diagram that summarizes the results of the literature search.

Initial database search resulted in 3,949 records. After excluding duplicates, 2,376 records were screened by title and abstract. Of these, 2,340 studies were not relevant and excluded. The remaining 36 records were assessed for eligibility. Notable exclusions were listed as follows: studies that did not use AA as an intervention (*n* = 6) (14–19), or not in patients with AP (*n* = 2) (20, 21), or clinical trials without a control arm (*n* = 1) (22). Two review/commentary articles were excluded (23, 24), five studies with no full text published (25–29) and two studies not published in English were excluded (30, 31). Four studies that did not report main outcomes of interest were excluded (32–35), and two studies reported overlapping data, so one of them was excluded (36). Finally, four studies were included in the meta-analysis for clinical trials (37–40) and nine articles (41–49) in the meta-analysis for preclinical studies (Figure 1).

### Study Characteristics

The four clinical trials involved a total of 219 patients, of whom 104 received AA administration, compared to 115 controls. Table 1 provides the details of the four clinical trials. The study by Siriwardena et al. was undertaken in the UK (37), the study by Du et.al. was undertaken in China (40), and the studies by Bansal et.al. and Sateesh et.al. were undertaken in India (38, 39). The study by Siriwardena et.al. was multicentered (37), the other three studies were single-centered. Studies by Sateesh et.al. and Du et.al. included patients with AP (39, 40) and studies by Siriwardena et.al. and Bansal et.al. included patients with predicted severe AP (37, 38). A study by Du et.al. prescribed AA as a monotherapy (40) and the other three studies used AA in combination with other antioxidant agents. AA was administrated orally in the study by Sateesh et.al. with a dose of 500 mg/day (39) and intravenously in other three studies with the dose ranging from 1 to 10 g/day. Three studies reported the time at which AA administration was initiated. In the study by Bansal et.al. AA was administrated within 96 h of disease onset (38), and in the studies by Siriwardena et al. and Sateesh et al. AA was administrated within 72 h (37, 39). The duration of AA administration ranged from 5 days to 2 weeks among the included studies.

**Table 1:** Characteristics of clinical trials included in the meta-analysis.

**Study**	**Country**	**No. of patients**	**AP severity**	**Intervention**	**Ascorbic acid regimen**	**Control**	**Initiation timing**
Du et al. (40)	China	40/44 (intervention/control)	AP	Ascorbic acid	Ascorbic acid 10 g/day, iv, for 5 days	Ascorbic acid 1 g/day, iv, for 5 days	Not mentioned
Siriwardena et al. (37)	UK	22/21 (intervention/control)	predicted severe AP^a^	Selenium, ascorbic acid, N-acetylcysteine	Day 1, 2: 2,000 mg/day, iv Day 3–7: 1,000 mg/day, iv	Placebo	Within 72 h of admission
Sateesh et al. (39)	India	23/30 (intervention /control)	AP	Ascorbic acid, N-acetyl cysteine, antoxyl forte	Ascorbic acid 500 mg/day by oral, for 5 days	No treatment	Within 72 h of developing symptoms
Bansal et al. (38)	India	19/20 (intervention /control)	Predicted severe AP^b^	Ascorbic acid, vitamin E, vitamin A	Ascorbic acid 1,000 mg/day, iv, for 14 days	No treatment	Within 96 h of developing symptoms

a *Predicted severe acute pancreatitis: an APACHE II score of 8 or more either at admission or within 48 h of admission.*

b *Predicted severe acute pancreatitis: an APACHE II score of 8 and CTSI ≥7.*

*AP, acute pancreatitis; iv, intravenously; APACHE II, Acute Physiology and Chronic Health Evaluation II; CTSI, computed tomography severity index*.

The nine preclinical studies included 11 experiments involving a total of 172 AP animals, of which 86 were controls and 86 had AA intervention. Table 2 details the characteristics of the nine included preclinical studies. The animal models adopted in these experiments mainly included caerulein-induced AP model (*n* = 2) (42, 48), retrograde infusion of sodium taurocholate-induced AP model (*n* = 1) (47), L-arginine-induced AP model (*n* = 4) (41, 44, 45), choline-deficient, ethionine-enriched (CDE) diet-induced AP model (*n* = 2) (49), and ischemia/reperfusion-induced AP model (*n* = 2) (43, 46). AA was administrated as monotherapy in seven experiments (43–45, 47–49) and in combination with other antioxidants in four experiments (41, 42, 46). The administration dose ranged from 30 μg/kg to 900 mg/kg body weight. AA was used prophylactically in three experiments (42, 46, 49) and therapeutically in eight experiments (41, 43–45, 47–49).

**Table 2:** Characteristics of preclinical studies included in the meta-analysis.

**Study**	**Animal model**	**Interventions**	**Ascorbic acid regimen**	**Initiation timing**
Nonaka et al. (49)	Female BALB/c mice with choline-deficient, ethionine enriched (CDE) diet induced AP model	A synthetic ascorbic acid derivative, CV3611	CV3611, 10 mg/kg, subcutaneously	12 h before or after CDE diet
Nonaka et al. (48)	Female BALB/c mice with caerulein-induced AP model	A synthetic ascorbic acid derivative, CV3611	CV3611, 10 mg/kg, subcutaneously	Just after the first caerulein injection
Hardman et al. (41)	Male Sprague-Dawley (SD) rats with L-arginine-induced AP model	Selenium, Ascorbic acid, N-acetylcysteine	Ascorbic acid 30 μg/kg, iv	6 or 24 h after AP induction
Esrefoglu et al. (42)	Female Wistar rats with two injection of caerulein at 2-h intervals induced AP model	L-ascorbic acid, N-acetyl Cysteine	Ascorbic acid 14.3 mg/kg, ip	Before AP induction
Sagiroglu et al. (44)	SD rats with L-arginine-induced AP model	Ascorbic acid	Ascorbic acid 200 mg/kg, iv	24 h after AP induction
Sidhu et al. (45)	Wistar rats with L-arginine-induced AP model	Ascorbic acid	Ascorbic acid 900 mg/kg, ip	Two hours after AP induction
Kochar et al. (46)	Male SD rats with ischemia/reperfusion-induced AP model	Ascorbic acid, Vitamin E	Ascorbic acid, ip, 200 mg/kg for 3 days	Before ischemia/reperfusion
Abogresha et al. (43)	Male SD rats with renal ischemia induced AP model	Ascorbic acid	Ascorbic acid 100 mg/kg, by oral	Right after AP induction
Xu et al. (47)	SD rats with retrograde infusion of a 5% sodium taurocholate solution induced SAP model	Ascorbic acid	Ascorbic acid 500 mg/kg, iv	Right after AP induction

*The animal numbers in each group in the study Sidhu et al. (45) were not reported, and we took 6 as a default value.*

*AP, acute pancreatitis; SAP, severe acute pancreatitis; CDE, choline-deficient, ethionine enriched; SD, Sprague-Dawley; iv, intravenously; ip, intraperitoneally*.

### Risk of Bias Assessment

**Figure 2:**
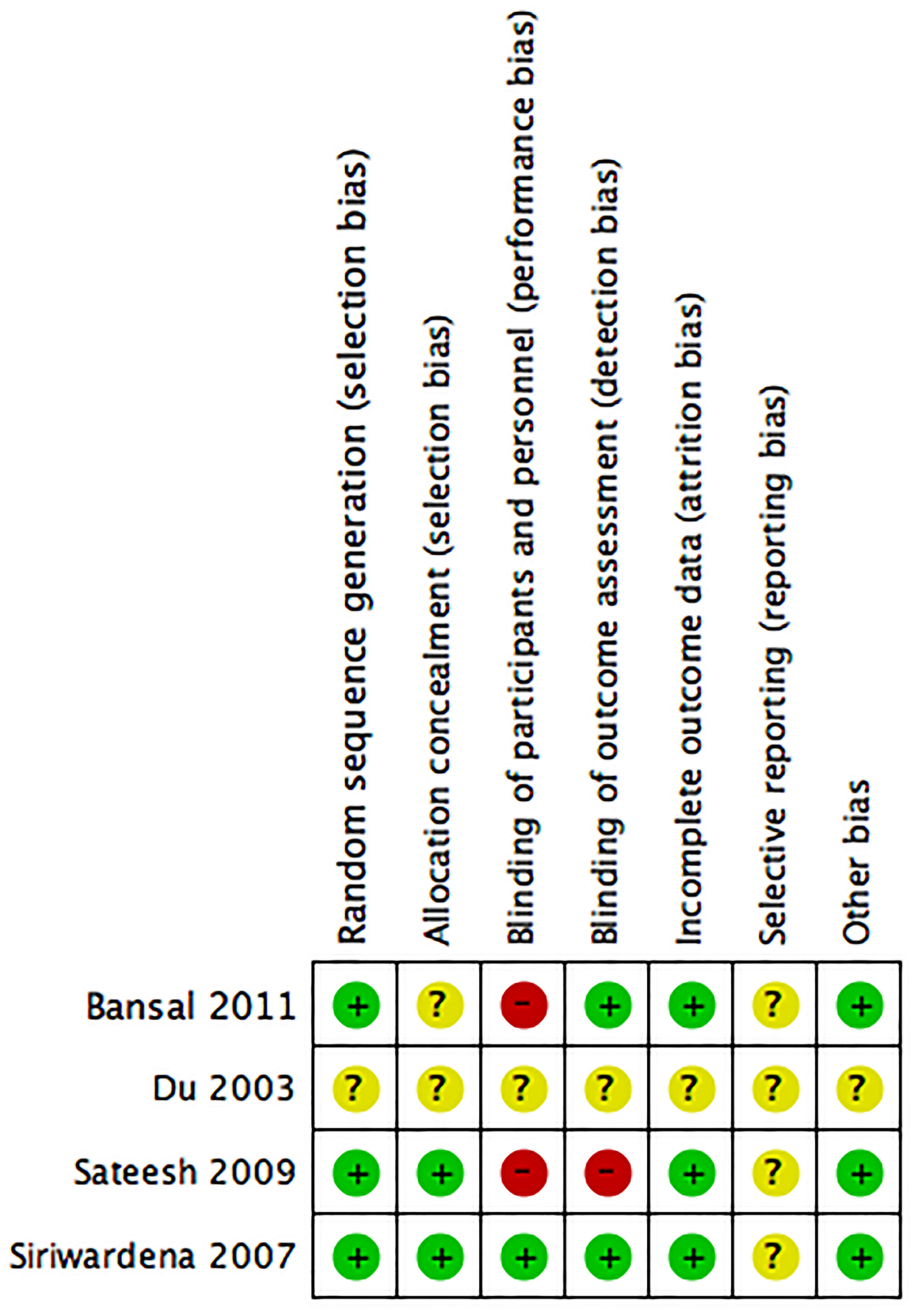
Risk of bias assessment of four clinical trials included in the meta-analysis.

For the clinical studies, the risk of bias assessment is presented in Figure 2. Studies by Siriwardena et al. Bansal et al. and Sateesh et al. were RCTs, with only the study by Siriwardena et.al. reporting the blinding of participants, physicians, and outcome assessment (37). Placebo was used in only the study by Siriwardena et al. (37). The study by Du et al. (40) did not report the methods in sufficient detail to draw conclusions about the risk of bias assessment.

None of the included preclinical studies reported on any of SYRCLE risk of bias tool items. Thus, the risk of bias was unclear or high in all studies.

### Meta-Analysis

#### Clinical Trials

**Figure 3:**
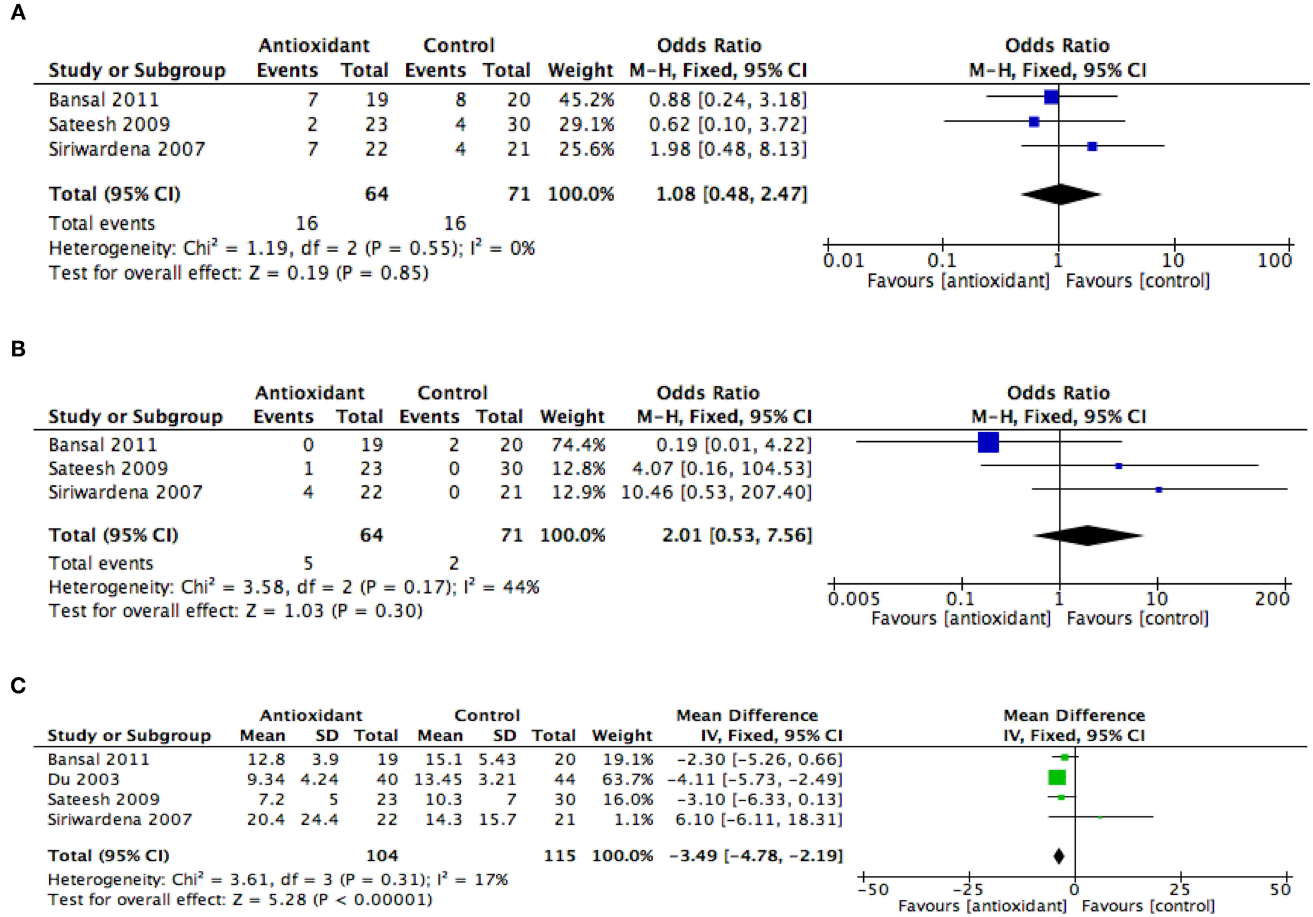
Summary meta-analysis of studies reporting effects of ascorbic acid administration on clinical outcomes in patients with AP compared with controls: **(A)** organ failure, **(B)** mortality, and **(C)** length of hospital stay. M-H, Mantel–Haenszel; SD, standard deviation; Std., mean difference, standardized mean difference; IV, inverse variance; CI, confidence interval; Tau^2^, tau-square statistic; Chi^2^, chi-square statistic; df, degrees of freedom; *I*^2^, I-square heterogeneity statistic; Z, Z statistic.

Three studies (37–39) involving a total of 135 patients were included in the meta-analysis of organ failure. Results from this analysis showed that there was no difference in the odds of developing organ failure in patients with AP administered with AA when compared with controls (OR 1.08, 95% CI 0.48–2.47, *p* = 0.85) (Figure 3A). The data among these three studies were found to have no heterogeneity (*I*^2^ = 0%).

Three studies (37–39) involving a total of 135 patients with AP were included in the meta-analysis of hospital mortality. Results from this analysis showed that AA administration in patients with AP did not significantly reduce hospital mortality when compared with controls (OR 2.01, 95% CI 0.53–7.56, *p* = 0.30) (Figure 3B). The study heterogeneity was found to be moderate (*I*^2^ = 44%).

All four studies involving a total of 219 patients were included in the meta-analysis of the length of hospital stay. Results from this analysis showed that AA administration in AP patients was associated with a reduction in the length of hospital stay when compared with controls (MD −3.49, 95% CI −4.78, −2.19, *p* < 0.001) (Figure 3C). The study heterogeneity was found to be low (*I*^2^ = 17%).

#### Preclinical Studies

**Figure 4:**
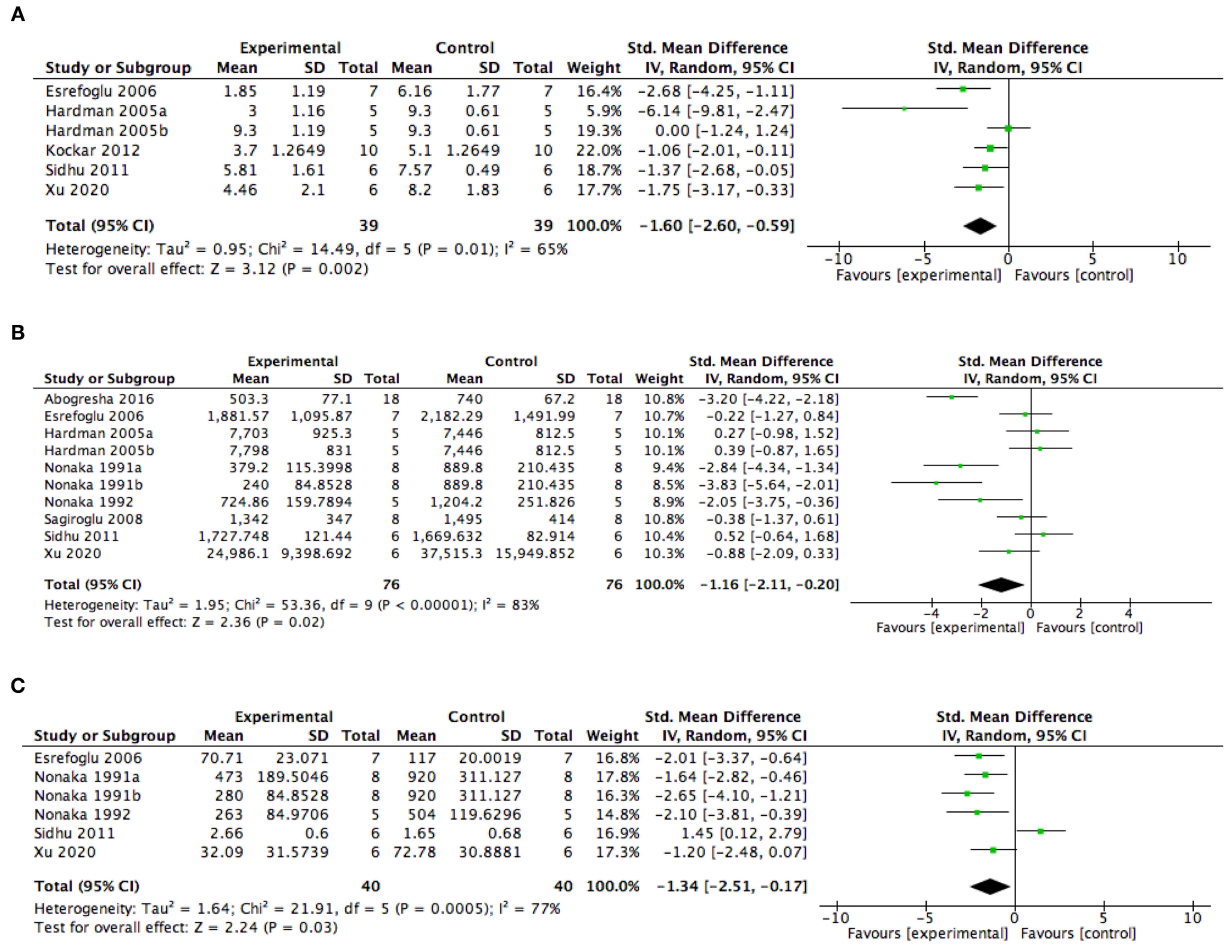
Summary meta-analysis of studies reporting the effect of ascorbic acid administration on outcomes in animal models with AP compared with controls: **(A)** histopathological scores of pancreatic injury, **(B)** serum amylase levels, and **(C)** serum lipase levels. SD, standard deviation; Std., mean difference, standardized mean difference; IV, inverse variance; CI, confidence interval; Tau^2^, tau-square statistic; Chi^2^, chi-square statistic; df, degrees of freedom; *I*^2^, I-square heterogeneity statistic, Z, Z statistic.

Meta-analysis of five studies (41, 42, 45–47) including six experiments was undertaken to examine the effect of AA intervention on pancreatic injury in animal models with AP. Results from this analysis showed that AA administration was associated with a reduced histopathological score of pancreatic injury (SMD −1.60, 95% CI −2.60, −0.59, *p* = 0.002) (Figure 4A). The study heterogeneity was found to be high (*I*^2^ = 65%).

Pertaining to physiological measures reflecting the severity of the pancreatic injury, eight studies including 10 experiments reported serum amylase levels and five studies including six experiments reported serum lipase levels. The meta-analysis showed that AA intervention significantly reduces the serum amylase levels (SMD −1.16, 95% CI −2.11, −0.20, *p* = 0.02) (Figure 4B) and the serum lipase levels (SMD −1.34, 95% CI −2.51, −0.17, *p* = 0.03) (Figure 4C) in AP animal models. But, the data among the included studies were highly heterogenetic.

## Discussion

This meta-analysis of clinical trials shows that AA supplementation, alone or in combination with other antioxidants, has no effect on survival and organ failure but is associated with a significantly reduced length of hospital stay. In preclinical studies, AA reduced pancreatic injury, but this result needs to be interpreted with caution, given the high heterogeneity of the included studies.

This is the first comprehensive meta-analysis systematically assessing the effects of AA supplementation on main outcomes of AP, both in clinical and preclinical studies. We focused on some important clinical outcomes, such as organ failure and mortality, and the results of our meta-analysis were in accordance with other studies in critically ill patients (50) concerning in-hospital mortality. Specifically, AA administration was associated with no significant effect on survival and neither supporting or discouraging the use of AA in patients with AP. However, a reduction in length of hospital stay (mean reduction of 3.5 days) was found with AA supplementation in AP. A similar benefit was also observed in other critically ill patients (51), which implies that AA may promote the recovery of patients with AP despite failing to reduce the incidence of inflammatory response or organ failure. This indicates some potential clinical and economic benefits of AA use in patients with AP.

Although the pathogenesis of AP is not fully understood, previous research indicated that oxidative stress and inflammatory response play important roles in it (52). The elevated oxidant levels (e.g., ROS) and decreased antioxidant defenses will cause lipid peroxidation, attack the biomembrane, and eventually lead to pancreatic acinar cell injury (53). In addition, evidence showed that ROS could promote the activation of NLRP3 inflammasome and induce systematic inflammatory responses (54). AA, as an important component in endogenous antioxidant defense, can directly scavenge ROS and other oxidative substances. It also modulates the inflammatory response by enhancing the phagocytic activity of leucocytes and inhibiting the activation of nuclear factor kappa B (NF-κB) pathways (7, 55). In our meta-analysis, AA supplementation could remarkably reduce the severity of AP in animal models, which was mainly demonstrated as alleviated pancreatic injury.

In exploring the clinical effects of AA on AP, the existing studies have small sample sizes, are mostly single-centered, and few are RCTs. It is also notable that the studies had different treatment regimens of AA, with varying doses, route of supplementation (orally or intravenously), bolus or continuous administration, initiation timing, duration, and alone or in combination with other antioxidant drugs. This variability prevents any definitive recommendation regarding the best way to deliver AA in the research and clinical settings. Hence, evidence to date remains sparse, scattered, and of suboptimal quality to draw firm conclusions on the effects of AA in patients with AP. Furthermore, a common limitation to all the studies was the inability to rapidly determine serum AA levels, which would allow titration of dose to know the level and achieve a defined goal. However, this lack of studies in the field coupled with the encouraging findings from this meta-analysis is of sufficient interest to justify further larger clinical trials.

The recommended daily intake of AA is ~100 mg in healthy individuals, producing a plasma concentration ranged from 60 to 100 μmol/L (56). Dietary AA is absorbed *via* the saturable sodium-dependent vitamin C transporter 1 (SCVC1) distributed in the gut (5), and the oral bioavailability (the amount of nutrient that will enter systemic circulation after oral intake) of AA in healthy people is variable and dependent on dose. With the increase in dose, the oral bioavailability decreases from almost 100% in low dose (<200 mg) to nearly 30% in high dose (e.g., 1,250 mg) (57). With sufficient AA dosing (100–300 mg/day), the plasma AA levels plateau at 70–85 μmol/L and do not exceed 220 μmol/L at maximal dosing (3 g/day) (58). Enteral intake is unpredictable and is seriously limited by saturable vitamin C transporter, and high oral intake may cause diarrhea (59). In addition, gut function often is impaired in patients with AP (60), especially in critically ill patients, which will decrease the absorption of AA (61). Hence, intravenous AA supplementation is preferred to enteral intake in severe critical illness patients like those with AP.

Intravenous administration of AA generally produces a predictable plasma concentration by avoiding limited and variable absorption, resulting in 100% bioavailability (5). Clinical pharmacokinetics studies indicated a linear relationship between dose and concentration of AA for doses up to 70 g/m^2^, resulting in a maximal plasma concentration of about 50 mmol/L (62, 63). Previous dose analysis studies in critically ill patients showed that normalization of plasma AA levels occurring only at 2–3 g/day by intravenous supplementation (64, 65). While direct antioxidant scavenging effects exist at markedly supraphysiologic levels (about 1–10 mmol/L, 10–100 times higher than the normal levels) (66), highlighting the use of high-dose (10–16 g/day) AA administration (65, 67, 68). In a phase I study on high-dose intravenous AA for patients with sepsis, a total dose of 200 mg/kg/day, given in four infusions per 6 h, rapidly led to plasma AA concentrations higher than 1,000 μmol/L (67).

Whether the dosing strategy of AA should aim at reaching normal or supraphysiologic levels still remains under debate. In the only dose-effect study in septic patients, the higher dose of 200 mg/kg/day intravenous AA showed greater effects on improving organ dysfunction than the lower dose of 50 mg/kg/day (67). In a recent meta-analysis carried out in critically ill patients, medium dose (3–10 g/day) AA has a positive role in mortality, which is not achieved by low (<3 g/day) or high doses (≥10 g/day) (69). There has been no consensus achieved on the optimal dose of AA in critically ill patients, let alone in patients with AP. It appears that at least 2–3 g intravenous AA must be supplemented daily during the acute phase to normalize plasma concentrations.

There has been no clinical trial exploring the optimal timing and duration of AA supplementation. In view of the ubiquitous AA depletion status in critically ill patients (70), including the patient with AP, it is appropriate to restore plasma AA levels as soon as possible after the primary disease insult, when oxidative stress is maximal (4). The optimal duration may differ between patients and depends on the severity of the disease. Previous studies warned that prolonged (i.e., several weeks or up to months) supplementation of high-dose AA should be avoided, which may increase the risk of oxalate nephropathy and oxalate kidney stone formation (71). In recent controlled studies, these complications have not been observed in patients with high doses for a short period (72), and renal function even improved (73). From the physiological perspective, some authors recommend that high-dose AA should be stopped after the acute phase of the disease (e.g., the first week in the disease course of AP) to allow the beneficial signaling function of oxidative substances, which are necessary for cell survival (4).

A pharmacokinetics clinical study carried out in critically ill patients by de Grooth et al. (65) showed that bolus dosing of AA provided a rapid plasma peak concentration while continuous dosing was effective in achieving relatively high steady-state concentrations. There has been no study directly comparing the superiority between bolus and continuous dosing till now. While the antioxidant scavenging effects of AA are dose-dependent, the transient high peaks might be beneficial. In a recent review (55), the clinically observed response to AA appeared to be attenuated when the daily dose was administered as a continuous infusion compared to bolus infusion. Other effects of AA, such as immune modulation are also dose-dependent (74).

In the case of bolus dosing, the optimal infusion interval is not known. Several pharmacokinetic studies of high-dose AA administration have calculated a constant elimination half-life of about 2 h following the discontinuation of intravenous infusion (62, 63, 75). This suggests that it takes about 8 h for a 1,000-μmol/L plasma concentration to become normalized to physiological levels. Hence, it seems reasonable to provide three to four bolus infusions in 24 h.

In this systematic review and meta-analysis, the lack of demonstrable effect of AA on survival and organ failure may temper enthusiasm for the use of AA in patients with AP, while it is premature to draw firm conclusions on the clinical effects of AA. Further higher quality studies are required, especially multicenter RCTs carried out in patients with predicted severe AP.

## Conclusion

Ascorbic acid deficiency is common in patients with AP and appears to be highly correlated with disease severity. A definite conclusion about the benefits of AA therapy in AP is not possible because of the relatively low quality of existing studies and the variation in study design. The meta-analysis of clinical trials does not show any improvement in survival or the rate of organ failure. However, it shows that AA administration was associated with a significantly reduced length of hospital stay for patients with AP. The meta-analysis of the preclinical trials showed that AA intervention is associated with reduced pancreatic injury. Taken together, these data provide sufficient evidence to justify higher quality trials to test the clinical benefits of AA, with study designs that seek to provide optimal dose, route, timing, and duration of AA administration in patients with AP.

## Data Availability Statement

The original contributions presented in the study are included in the article/supplementary material, further inquiries can be directed to the corresponding author/s.

## Author Contributions

LG and JW drafted the work and revised it critically for intellectual content. LG and EC made a substantial contribution to the acquisition and interpretation of data. SP and AP revised it critically for important content. LK and WL made substantial contributions to the conception and design of the work. All authors have read the manuscript and approved its submission.

## Funding

This work was supported by the National Natural Science Foundation of China (No. 82070665) and Applied Basic Research Project of PLA (No. ALB19J002).

## Conflict of Interest

The authors declare that the research was conducted in the absence of any commercial or financial relationships that could be construed as a potential conflict of interest.

## Publisher's Note

All claims expressed in this article are solely those of the authors and do not necessarily represent those of their affiliated organizations, or those of the publisher, the editors and the reviewers. Any product that may be evaluated in this article, or claim that may be made by its manufacturer, is not guaranteed or endorsed by the publisher.
